# Antimicrobial and antifungal activity of soil actinomycetes isolated from coal mine sites

**DOI:** 10.1016/j.sjbs.2021.03.029

**Published:** 2021-03-17

**Authors:** Kasarla Sarika, Gattu Sampath, Rasiravathanahalli Kaveriyappan Govindarajan, Fuad Ameen, Suaad Alwakeel, Hussah I. Al Gwaiz, Thampu Raja Komuraiah, Gangalla Ravi

**Affiliations:** a Department of Microbiology, Kakatiya University, Warangal 506009, Telangana, India; b Department of Zoology, School of Life Sciences, Periyar University, Salem 636011, Tamil Nadu, India; c Division of Biotechnology, School of Agro-Industry, Faculty of Agro-Industry, Chiang Mai University, Chiang Mai 50100, Thailand; d Department of Botany and Microbiology, College of Science, King Saud University, Riyadh 11451, Saudi Arabia; e Department of Biology, College of Science, Princess Nourah Bint Abdulrahman University, Riyadh 11564, Saudi Arabia

**Keywords:** Coalmine soil, Molecular approach, Streptomyces sp, Crude extract, Antimicrobial activity

## Abstract

In the current study, twenty-eight soil samples were collected from coalmine sites of Telangana, India. The isolates were purified and identified based on their culture characterization on oatmeal agar, glycerol asparagine agar, yeast extract-malt extract agar, inorganic salt starch agar, and starch casein agar medium. Further, the supernatant of all the isolates were tested for antimicrobial and antifungal activities. The biochemical and microscopic studies of isolated strains results indicates the potential isolate strains belongs to Streptomyces genus. Among all the strains the biological activity of BHPL-KSKU5 showed higher anti-bacterial and anti-funagal activity. The molecular characterization of BHPL-KSKU5 16s rDNA gene sequence and phylogenetic tree showed that is mostly related to the *Streptomysis felleus* (*S. felleus*) strain. This isolate was submitted to gene bank NCBI with accession number MH553077. In addition, physiological studies such as utilization of carbon, nitrogen, amino acid sources of potential isolated were studied. Further, optimization, purification and characterization of the novel compound producing strain may be helpful for discovering the new therapeutic microbial agent.

## 1 Introduction

Microbial diversity is a major frontier and future source for the biotechnology sector (Pramanik et al., 2019). Microorganisms produced natural products that are a good source of antibiotics, including actinomycetes (Karthik et al., 2020, Srivastava et al., 2019). Actinomycetes are gram-positive and slow-growing bacteria, distinguished by the development of aerial mycelium. They make mycelium from spores that anchor the substrate (Williams et al., 1983). In the vegetative process, the substratum hyphae have a diameter of around 0.5 to 1.0 μm and lack cross-walls. Actinomycetes have acquired prominence in recent years because of their antibiotic capacity. Actinomycetes are a group of microbes widely distributed across the world's natural ecosystems and are especially valuable for their organic cycling role (Currie et al., 2006, Javed et al., 2020). Actinomycetes have unique bioactive metabolites, including antibiotics, enzymes, and plant growth factors (Nanjwade et al., 2010, Dimri et al., 2020, Gunasinghe and Edirisinghe, 2020). The development of multi-drug-resistant pathogens requires unique antimicrobial agents (Spellberg et al., 2013), at least 5000 known Streptomyces sp. (*Streptomyces avermitilis* and *Streptomyces verticillus*) were produced bioactive compounds. A broad spectrum of antibiotic products like macrolides, β-lactams, polyenes, aminoglycosides, tetracyclines, peptides, and polyethers are produced by most of the Streptomyces sp (Augustine et al., 2005). Several studies showed Actinomycetis have wast range of biomedical applications such as antibacterial (Kurnianto et al., 2020), antibiofilm (Mulya and Waturangi 2021), antifunagal (Wei et al., 2020), and anticancer (Rajivgandhi et al., 2020) activities.

Coalmine soil abounds conatain a broad range of microbes, in whcich actinomycetes have great interest in finding new genera with novel bioactive compounds. Coal mine soil actinomycetes are regarded as sources of new antibiotics; anti-cancer agents and other secondary metabolites. Coal is called a fossil fuel because which is made up of materials that were once living plants and its possesses chemical components like carbon, hydrogen, sulfur, and nitrogen; therefore, it is suggested that one could isolate diverse (Thakur et al., 2012) range of actinomycetes from the coal mine soil, it is therefore important for a novel, bioactive secondary metabolites to be isolated by different types of coalmines from unexplored and underexplored environments (Singh et al., 2006). Streptomyces belongs to the genus of actinomycetes, which contain 70% of antibiotics and beneficial in many agriculture and food and pharmaceutical industry industries (Thakur et al., 2012, Govindarajan et al., 2021). A higher number of reports need to carry out to discover new antibiotics, which is essential to control the multidrug-resistant pathogenic microorganisms. The present study has been performed to illustrate the antimicrobial potential of Streptomyces isolated from Telangana, India's coal mine soils.

## 2 Materials and methods

### 2.1 Collection of soil sample

28 soil samples were collected from diffrent coalmine sites from Bellampally (BPL) 19°02'44.3''N 79°30'46.2''E, Bhupalpally (BHPL) 18°27'05.0''N 79°51''14.0''E, Godavarikhani (GDK) 18°42'45.0''N 79°31'48.4''E, Kothagudem (KGDM) 17°28'51.3''N 80°40'19.4''E, Sathupally (SPL) 17°12'18.2''N 80°47'39.6''E, Madhamari-Kalyankhani (MM-KK) 19°00'20.7''N 79°28'08.1''E, and Ravindhrakhani (RK) 18°53'17.9''N 79°30'04.9''E,Telangana, India, during 2019 using an open-end soil borer (10 cm in depth and 25 cm in diameter), then air-dried for 24 hr (Dezfully and Ramanayaka 2015). The soil samples were transferred to sterile polythene zip lock bags, and the samples were carried to the Microbiology laboratory of Kakatiya University, for further analysis.

### 2.2 Isolation of actinomycetes

Samples were allowed to 10 fold serial dilution with one gram of soil dissolved in sterile water and were inoculated on starch casein agar. Streptomycin (30 µg/L) and amphotericin B (50 µg/L) were added to the medium to retard bacteria and fungi growth, respectively. Later all the plates were incubated at 28 ± 2 °C for 4 to 10 days. The suspected colonies structures isolates with brown and white colonies were purified. The pure cultures were streaked on to respective medium plates.

### 2.3 Morphological studies

The pure isolates were prepared for actinomycetes plates and placed 3 to 4 sterile coverslips. The plates were incubated at 28 ± 2 °C for 4 to 7 days. The coverslips were removed at 3 days of interval and observed under the high power magnification. The arrangement of conidiospores on aerial and substrate mycelia was observed and compared with the Bergeys Manual of Determinative Bacteriology.

Cultural characteristics were conducted by growing the organism on oatmeal agar, glycerol asparagine agar, yeast extract-malt extract agar, inorganic salt starch agar, and starch casein agar media after 14 days of culturing at 28 °C. Morphological characteristics of aerial hyphae, spore chain, spore mass, spore surface, the colour of aerial and substrate mycelia, and diffusible pigments production were conducted by growing the organism on ISP-4 medium for 7-days and accessed via light microscope.

The culture plates containing actinomycetes were prepared, and coverslips were placed at an angle of 45 °C and allowed to incubate at 28 °C for 7 days, later coverslips were carefully removed and examined under a microscope. The aerial and substrate of conidiospores' mycelial structure have been attributed to the Bergeys Manual of Determinative Bacteriology (Arifuzzaman, et al., 2010; Williams et al., 1983).

### 2.4 Molecular characterizations of BHPL-KSKU5

To identify the isolate species name by molecular methods, 16S rDNA molecular analysis was performed. The potential strain BHPL**-**KSKU5 DNA was isolated, the single band of DNA was observed in 1.0% Agarose gel electrophoresis. Fragment of 16S rDNA gene amplification was performed by 27F and 1492R primers. Forward and reverse DNA sequencing reaction of PCR amplicon was carried out with forward primer and reverse primer using BDT v3.1 Cycle sequencing kit on ABI 13730xlGenetic Analyzer. The consensus sequence of the 16S rDNA gene was generated from forward and reverse sequence data using aligner software, the phylogenetic tree was constructed using MEGA7 software.

### 2.5 Biochemical studies

Utilization of Carbon and Nitrogen sources

The Bennett broth was produced from disparate sources of carbon such as L-arabinoses, D-arabinoses, maltoses, mannose, dextrose, starch, D-sorbitol, sucrose, lactose, D-galactose, fructose, and xylose was autoclaved followed by tubing individually. Starch casein broth containing nitrogen components like ammonium nitrate, ammonium sulphate, potassium nitrate, sodium nitrate, calcium nitrate, asparagine, and the urea were added individually. Actinomycetes colonies were then incubated for 15 days in every container comprising broth, which contained subsequent tubes at 28 °C and incubated after the growth was recorded.

### 2.6 Utilization of amino acids

Modified Bennett broth was prepared with sources such as DL-2 amino-N-butric acid, L-tyrosine, DL-tryptophan, DL-ornithine, L-lysine, L-glutamic acid, L-cystein, DL-aspartic acid, DL- leucine, L-arginine, DL-alanine, and L-leucine. The Streptomyces culture was inoculated into each tube containing the broth. Then the tubes were incubated at 28 ± 2 °C for 15 days. After incubation the growth pattern was recorded.

### 2.7 Bacterial cultures

Five different gram-positive bacteria *Staphylococcus aureus* (*S. aureus*), *Baciilus cereus* (*B. cereus*), *Streptococcus pneumonia* (*S. pneumonia*), *Bacillus subtilis* (*B. subtilis*) and *Micrococcus luteus* (*M. luteus*) and five gram-negative bacteria *Enterobacter aerogenes* (*E. aerogenes*), *Klebseilla pneumonia* (*K. pneumonia*), *Escherichia coli* (*E. coli*), *Salmonella paratyphi* (*S. paratyphi*) *Pseudomonas aeruginosa* (*P. aeruginosa*) and *Candida albicans* (*C. albicans*) were screened against antimicrobial compound produced by actinomycetes isolates. The tested bacteria were obtained from the department of Botany and Microbiology, King Saud University, Riyadh, Saudi Arabia and Microbiology laboratory, Kakatiya Medical College, Warangal, India.

### 2.8 Extraction of crude antimicrobial metabolite and antimicrobial activity, anti-candidal activity

The ethyal acetate crude extract was derived by inoculating Streptomyces strain in starch casein broth and incubated in a shaker at 28 ± 2 °C, 200 rpm for 4–8 days. A single-centre streak was made by the potent actinomycetes isolates on nutrient agar medium plates and incubated at 28 ± 2 °C for 4 days. The potent actinomycetes strains were inoculated into the 250 mL Erlenmeyer flasks containing Starch casein broth and incubated in an orbital shaker at 200 rpm at 28 ± 2 °C for 7 days. After incubation, cultures were subjected to centrifugation at 10,000 rpm for 10 min. The supernatant was aseptically collected, and their antimicrobial efficacy (900 μg/mL) was tested by employing agar well diffusion assay, the crude obtained from all the isolates was carried on against the test pathogens namely *B. subtilis, B. cereus, S. aureus, S. pneumoniae, M. luteus*, *E. coli, E.aerogenes, K. pneumoniae, S. paratyphi* and *P.aeruginosa*. Muller Hinton agar plates were prepared and lawn culture of bacteria was made. The nutrient broth was cultivated on bacterial sample organisms for 24hr. For the preparation of the bacterial lawn, a 100 mg cultivation of each bacterial species was used (1 × 10^-5^ CFU/mL). Agar wells of 6 mm diameter were prepared with the help of a sterile cork borer. The wells were loaded with crude extract 900 µg/mL, negative control (DMSO), along with 30 µg/mL of streptomycin as a positive control. The plates were incubated 24 hr at 37 °C and the zone of inhibition was calculated (Dhanasekaran et al., 2008).

Approximately 50 mL of potato dextrose agar was poured onto the sterilized petri plate utilizing the agar well diffusion assay to assay the anti-candidal activity (Kengpipat et al., 2016). The *C. albican* testing fungus was conducted in a saline solution (0.85%), NaCl medium, and customized to 0.5 Mc Farland and (10^-8^ CFU/mL) turbidity. A sterilized spreader was used to spread 1 mL of the sample culture into PDA plates. Agar wells of 6 mm diameter were prepared. The wells were loaded with crude extracr (900 µg/mL), negative and positive control after the plates were incubated for 48 hr. The inhibition zone diameter was measured in millimetre. Based on the size of the zone of inhibition, the potent antibacterial compound contains isolate were selected.

### 2.9 Statistical data analysis

The data were evaluated using (SPSS V.10.0) and Excel. All the data expressed as mean ± standard deviation (SD) for each test for triplicate.

## 3 Results

**Fig. 1:**
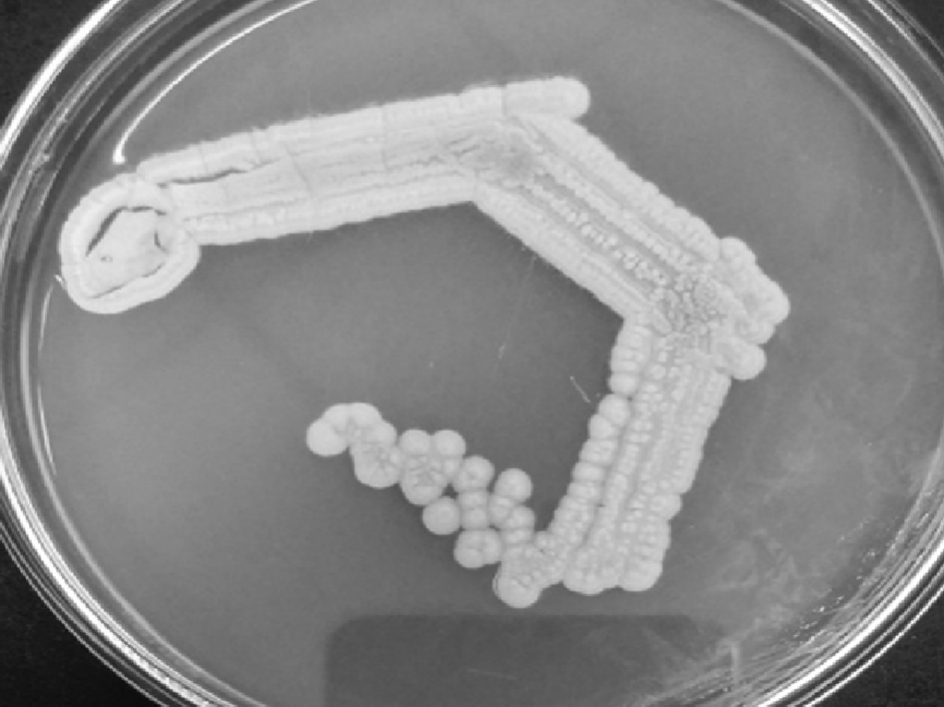
Morphology view of isolate of BHPL- KSKU5 growth in Glycerol asparagine agar.

A total 28 soil samples were obtained from various coalmine soil samples of Telangana, India. From these actinomycetes colonies (morphologically different) were purified by sub-cultured and stored at 4 °C and used for further studies. The isolates aerial spore mass colour was categorized into 3 groups grey, ash, and white series (Fig. 1). Most of the isolates produced grey and white series than ash series.

### 3.1 Extraction of crude antimicrobial compounds

The fermented broth containing antimicrobial compounds of selected potential Streptomyces was extracted by centrifugation. All the crude extracts were used for antimicrobial activity.

### 3.2 Screening of antimicrobial compound producing actinomycetes

All the isolates exhibited antimicrobial and anti fungal activity (Table. 1) against tested bacteria with varying inhibitory zones. Among them, only BHPL**-**KSKU5 isolate showed a broad range of antimicrobial activity, which showed higher zone of inhibition against *S. paratyphi* and *C. albicans* (18 ± 0.4 mm and 16. ± 0.4 mm) hence, the BHPL**-**KSKU5 was selected for further investigations.

**Table 1:** Screening of antimicrobial activity of actinomycetes isolates. Results were expressed as the mean ± SD of three individual experiments, NA = No activity.

Zone of inhibition (mm)											
Isolate code	*B.subtilis*	*B.cereus*	*S.aureus*	*S.paratyphi*	*M.luteus*	*E.coli*	*E.aerogenes*	*K.pnuemoniae*	*S.paratyphi*	*P.aeruginosa*	*C.albicans*
BPL-KSKU1	5.1 ± 0.3	7 ± 0.5	5 ± 0.4	8. ± 0.4	3.9 ± 0.1	8 ± 0.4	8 ± 0.0	5 ± 0.4	10 ± 0.3	9 ± 0.2	8 ± 0.6
BPL-KSKU2	NA	NA	9 ± 0.15	6.1 ± 0.3	3 ± 0.2	5.9 ± 0.7	NA	7.8 ± 0.7	9 ± 0.25	7 ± 0.3	6 ± 0.2
BPL-KSKU3	7 ± 0.3	NA	9 ± 0.25	9 ± 0.3	NA	4.9 ± 0.3	7.9 ± 0.4	10 ± 0.6	8 ± 0.2	NA	NA
BPL-KSKU4	12 ± 0.5	8.1 ± 0.6	NA	10 ± 0.4	4 ± 0.3	NA	5 ± 0.2	NA	6 ± 0.6	11 ± 0.5	NA
BPL-KSKU5	NA	6.2 ± 0.4	6.9 ± 0.5	8 ± 0.4	8 ± 0.4	8.9 ± 0.4	NA	7.9 ± 0.5	NA	NA	NA
BPL-KSKU6	6.1 ± 0.4	NA	9.9 ± 0.5	NA	NA	NA	6.1 ± 0.4	NA	3 ± 0.25	NA	NA
BHPL-KSKU3	5.9 ± 0.3	4.9 ± 0.5	3.9 ± 0.2	7.9 ± 1.3	3 ± 0.3	4 ± 0.4	7.9 ± 0.5	5. ± 0.4	9 ± 0.35	6 ± 0.8	8 ± 0.4
BHPL-KSKU5	**12 ± 0.4**	**6.9 ± 0.4**	**7.9 ± 0.4**	**18 ± 0.4**	**5.03 ± 0.2**	**9.1 ± 0.45**	**5 ± 0.39**	**9 ± 0.5**	**18 ± 0.3**	**8 ± 0.1**	**16 ± 0.4**
BHPL-KSKU19	9.6 ± 0.3	4 ± 0.3	8 ± 0.4	10 ± 0.4	9 ± 0.2	11 ± 0.4	8.1 ± 0.26	5.9 ± 0.2	13.9 ± 0.25	10 ± 0.3	9 ± 0.2
BHPL-KSKU20	8.9 ± 0.5	5 ± 0.3	6.9 ± 0.3	6 ± 0.3	5.1 ± 0.3	10 ± 0.4	5.9 ± 0.7	8.03 ± 1.6	7.9 ± 0.3	6 ± 0.9	6 ± 0.2
BHPL-KSKU23	8 ± 0.3	6 ± 0.3	9.9 ± 0.2	12 ± 0.4	5.9±0.3	12 ± 2.1	10 ± 0.3	9 ± 0.4	17.9 ± 0.3	12 ± 0.3	9 ± 0.2
BHPL-KSKU25	5 ± 0.3	5.9 ± 0.3	8.9 ± 0.2	7.9 ± 0.2	4.9 ± 0.2	9.8 ± 0.32	8 ± 0.4	6.9 ± 0.3	11 ± 0.2	9 ± 0.3	8 ± 0.35
GDK-KSKU1	NA	5.1 ± 0.2	NA	6 ± 0.4	2.9 ± 0.4	NA	9.1 ± 0.5	NA	NA	5.9 ± 0.2	NA
GDK-KSKU2	4.9 ± 0.6	NA	5.03 ± 0.2	4.1 ± 0.26	NA	9.9 ± 0.2	7 ± 0.3	6 ± 0.3	4 ± 0.3	10 ± 0.2	NA
GDK-KSKU3	7 ± 0.3	8 ± 0.3	6.9 ± 0.4	8.9 ± 0.2	6 ± 0.4	12 ± 0.2	12.9 ± 0.2	10.1 ± 0.3	NA	8 ± 0.4	NA
GDK-KSKU4	NA	9 ± 0.2	NA	6 ± 0.3	NA	NA	9.9 ± 0.2	5 ± 0.3	8. ± 0.4	NA	1.9 ± 0.2
GDK-KSKU6	9.1 ± 0.3	4.9 ± 0.2	6.9 ± 0.1	9.9 ± 0.3	8 ± 0.4	12.1 ± 0.4	11 ± 0.15	8 ± 0.35	13.1 ± 0.26	9 ± 0.25	4.9 ± 02
GDK-KSKU12	9.9 ± 0.2	8 ± 0.3	7 ± 0.4	9.0 ± 0.2	5.9 ± 0.3	8.9 ± 0.3	7.9 ± 0.15	5. ± 0.4	11 ± 0.2	8 ± 0.4	5.5 ± 0.4
GDK-KSKU16	8 ± 0.15	6 ± 0.3	5 ± 0.3	11 ± 0.2	8.9 ± 0.1	10 ± 0.3	7 ± 0.4	9 ± 0.3	9.9 ± 0.4	5.9 ± 0.1	9 ± 0.3
KGDM-KSKU1	5.4 ± 0.5	NA	7 ± 0.2	7.8 ± 0.5	3 ± 0.4	2.1 ± 0.2	6 ± 0.35	8 ± 0.3	11.9 ± 0.7	7.9 ± 0.2	5 ± 0.7
KGDM-KSKU2	3.9 ± 0.2	6 ± 0.25	NA	8 ± 0.1	NA	7 ± 0.2	5 ± 0.6	NA	NA	6 ± 0.3	8 ± 0.3
KGDM-KSKU3	NA	8 ± 0.9	6.9 ± 0.2	3.9 ± 0.2	2 ± 0.2	5 ± 0.4	NA	6.8 ± 0.3	12.9 ± 0.2	8.9 ± 0.2	3 ± 0.2
KGDM-KSKU4	4.9 ± 0.2	4 ± 0.4	5 ± 0.3	5.9 ± 0.8	4 ± 0.3	8.9 ± 0	6.9 ± 1.1	5.9 ± 0.4	9 ± 0.3	3 ± 0.5	8.9 ± 0.4
KGDM-KSKU15	9 ± 0.2	6.9 ± 0.3	11.9 ± 0.1	9.9 ± 0.3	7.7 ± 0.6	5.9 ± 0.4	3.9 ± 0.2	9 ± 0.3	9.9 ± 0.2	8 ± 0.4	4.9 ± 0.2
MM-KK-KSKU9	5 ± 0.4	5 ± 0.5	9.9 ± 0.5	9.1 ± 0.6	7.1 ± 05	11 ± 0.5	8 ± 0.3	10 ± 0.2	16 ± 0.2	10 ± 0.3	9 ± 0.4
MM-KK-KSKU5	NA	7.1 ± 0.4	7.9 ± 0.5	4.9 ± 0.4	11 ± 0.4	8.1 ± 0.4	5 ± 0.5	11 ± 0.2	NA	NA	4.03 ± 0.4
SPL-KSKU25.1	5.1 ± 0.3	5.8 ± 0.4	9 ± 0.6	8.1 ± 0.5	9.9 ± 0.0	5.1 ± 0.4	9 ± 0.4	8.9 ± 0.7	12.1 ± 0.3	10 ± 0.4	5.9 ± 0.5
RK-KSKU1	3.1 ± 0.6	9. ± 0.3	4.1 ± 0.8	6.03 ± 0.6	9.1 ± 0.4	4 ± 0.1	6. ± 0.35	5.1 ± 2.1	2.1 ± 0.3	3 ± 0.8	9 ± 0.4
Streptomycin	15 ± 1.2	20 ± 0.5	22 ± 2.1	22 ± 1.8	19 ± 0.4	20 ± 0.1	18 ± 0.5	18 ± 0.7	19 ± 2.5	22 ± 3.1	NA
DMSO	NA	NA	NA	NA	NA	NA	NA	NA	NA	NA	NA

(Bellampally (BPL), Bhupalpally (BHPL), Godavarikhani (GDK), Kothagudem (KGDM), Sathupally (SPL), Madhamari-Kalyankhani (MM-KK), Ravindhrakhani (RK) and KSKU-KasarlaSarikaKakatiya University)

### 3.3 Identification of selected strain

The actinomycetes isolate BHPL**-**KSKU5 with broad-spectrum antimicrobial activities was selected for further characterization based on morphological, physiological, and molecular identification by 16S rDNA molecular analysis.

The microscopic observation under light microscopy revealed the BHPL**-**KSKU5 formed a flexuous spore chain on aerial mycelium (Table. 2). The microscopic studies of the isolates placed these isolates under Streptomyces genera. The carbon utilization of the potent strain BHPL**-**KSKU5 showed positive to dextrose, L-arabinose, maltose, starch, D-arabinose, fructose, mannose, and lactose but is unable to utilize mannitol, sucrose, xylose, and D-galactose (Table. 3). The utilization of different nitrogen sources was also studied among seven different nitrogen sources strain BHPL**-**KSKU5 showed positive results to sodium nitrate and asparagine whereas it didn’t utilize the ammonium sulphate, ammonium nitrate (Table. 3). The utilization of amino acids were also tested. The DL-2 amino-N-butric acid, L-tyrosine, DL-tryptophan, DL-ornithine, L-lysine, DL-leucine, L-arginine, DL-alanine, L-leucine were utilized. Where as L-glutamic acid, L-cystine, and DL-aspartic acid aren't used (Table.3).

**Table 2:** Cultural characteristics of potent actinomycetes on different culture media.

Name of the culture medium	*Streptomysis felleus* (BHPL-KSKU5)
Glycerol asparagine agar	
a) Aerial mycelium	Grey
b) Substrate mycelium	Dull white
c) Pigmentation	Nil
Yeast extract malt extract agar	
a) Aerial mycelium	Light grey
b) Substrate mycelium	Light brown
c) Pigmentation	Nil
Starch casein agar	
a) Aerial mycelium	Grey
b) Substrate mycelium	Dull white
c) Pigmentation	Nil
Tyrosine agar	
a) Aerial mycelium	Grey
b) Substrate mycelium	Dull white
c) Pigmentation	Nil
Oat-meal agar	
a) Aerial mycelium	White
b) Substrate mycelium	Dull white
c) Pigmentation	Nil

**Table 3:** Physiological studies of BHPL KSKU5.

S.No.	Name of the carbon sources	*Streptomysis felleus* BHPL-KSKU5
1	L-arabinose	+
	D-arabinose	+
	Dextrose	+
	Maltose	+
	Mannose	+
	Starch	+
	D-sorbitol	+
	Sucrose	–
	Fructose	+
	Lactose	+
	D-galactose	–
	Xylose	–
2	**Name of the nitrogen sources**	
	Ammonium sulphate	–
	Ammonium nitrate	–
	Sodium nitrate	+
	Potassium nitrate	+
	Calcium nitrate	+
	Aspargine	+
	Urea	–
3	**Name of the amino acid sources**	
	DL-2 amino-N-butric acid	+
	L-tyrosine	+
	DL-tryptophan	+
	DL-ornithine	+
	L-lysine	+
	DL-leucine	+
	L-arginine	+
	DL-alanine	–
	L-leucine	+
	L-glutamic acid	–
	L-cystine	–
	DL-aspartic acid	–

**Fig. 2:**
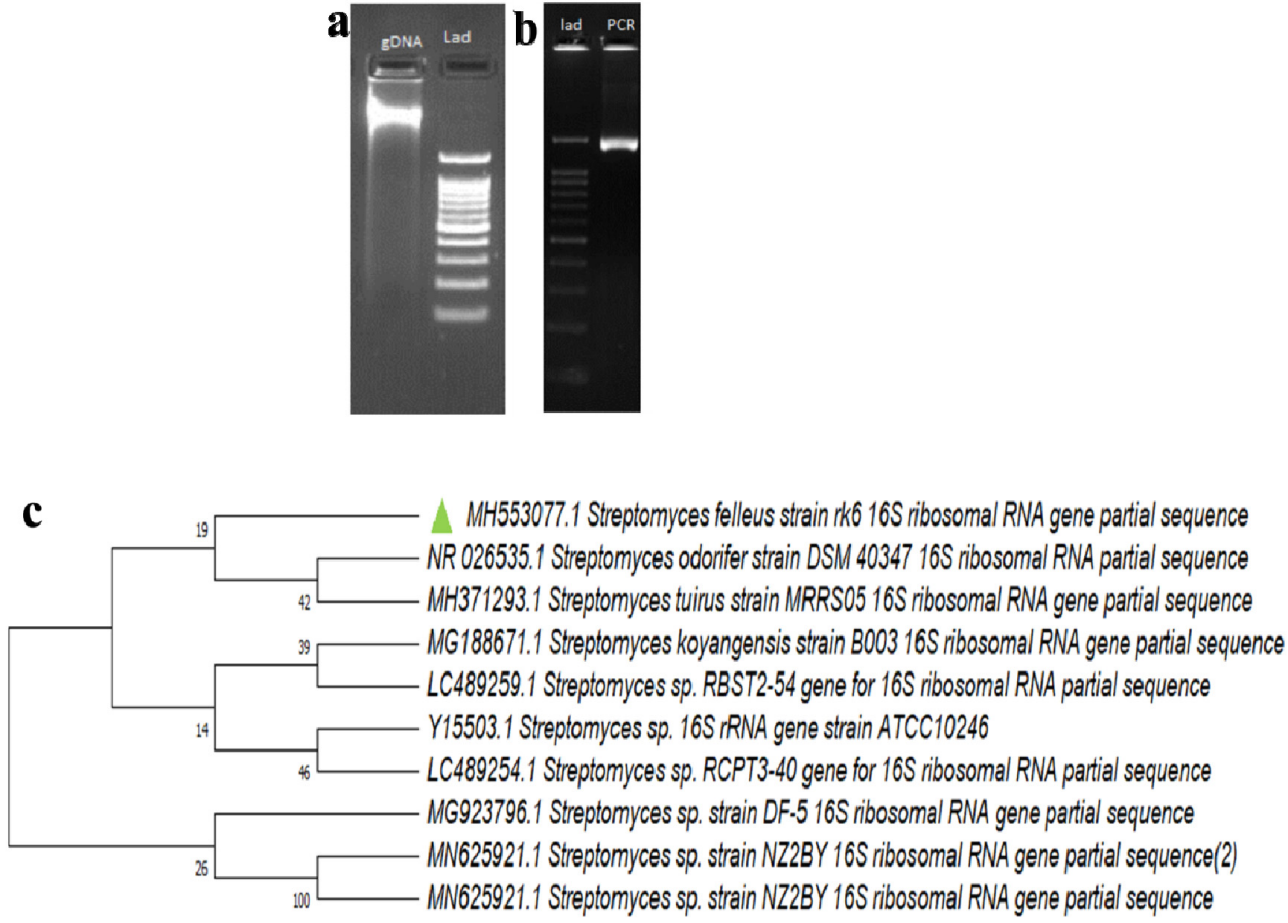
a) Confirmation of genomic DNA using 0.8% and b) amplified DNA PCR 16 s DNA using 1.0% gel c) construction of phylogenetic tree using neighbor-Joining method of strain BHPL- KSKU5 (MH553077 NCBI accession number) with other reference strains. Present identified strain was highlighted with Δ symbol.

The molecular characterization (Fig. 2**a, b**) was performed by PCR amplification. The 16S rDNA gene sequence was used for BLAST analysis. Based on the maximum similarity score, the first 10 sequences were used and aligned with multiple alignment ClusalW software. Using the MEGA 7 the distance matrix phylogenetic tree was developed. It revealed that BHPL**-**KSKU5 found 98% similarity with the existing species of *S. felleus* based on nucleotide homology and phylogenetic analysis (Fig. 2**c**). The sequence was submitted to Genebank and obtained accession number MH553077.

## 4 Discussion

Actinomycins produce the different types of bioactive compounds, which contain antibiotic, anticancer, and antimicrobial activities (Elsayed et al., 2020, Bukhari et al., 2021). Actinomycetes are omnipresent in the earth's solid constituents, such as humus, dung, litter, and soils. Actinomycetes are found in the air in which their hyphal biomass is dispersed through fragmentation and are carried by air and water and grow at the extent of the organic residues.

Actinomycetes have a significant property in digestion and production of certain components such as proteins in the form of keratin and certain vitamins (Lechevalier, 1981). A large majority of antibiotics that have been isolated in the numerous tested programs for new chemotherapic agents against various bacteria (Waksman et al., 1952). Two-third of the naturally available antibiotics were produced from actinomycetes (Tanaka and Omura, 1990). Streptomyces are specifically prolific and can make many biologically active secondary metabolites and antibiotics.

In the current study, actinomycetes isolate namely *S. felleus* (BHPL**-**KSKU5) was found to develop a flexuous spore chain with a smooth surface, this is the main characteristic feature of Streptomyces, these study was supported by Atalan et al. (2000). The composition of the medium is an essential aspect ofthe morphology of the microorganisms. The actinomycetes isolates were grown on different culture media like yeast extract malt extract agar, oatmeal agar, glycerol asparagine agar, tyrosine agar medium and starch casein agar (Ouchari et al., 2019), starch casein agar was best suitable than the other media tested.

For the characterization of actinomycetes isolates metabolites, various physiological tests were carried out (Abbas et al., 2019). In the current study the *S. felleus* (BHPL**-**KSKU5) showed positive results in carbon utilization to dextrose, L-arabinose, maltose, starch, D-arabinose, fructose, mannose, and lactose but it unable to utilize mannitol, sucrose, xylose, and D-galactose. The utilization of different nitrogen sources in strain BHPL**-**KSKU5 showed positive results to sodium nitrate and asparagine, whereas it did not utilize ammonium sulphate, ammonium nitrate but utilized potassium nitrate, and calcium nitrate. The utilization of amino acids was also tested. Among the tested amino acids, namely DL-2 amino-N-butric acid, L-tyrosine, DL-tryptophan, DL-ornithine, L-lysine, DL-leucine, L-arginine, DL-alanine and L-leucine were utilized. Whereas it did not utilize L-glutamic acid, L-cystine, and DL-aspartic acid, these results could be utilized as a taxonomic criterion at genus level identification.

In identifying actinomycetes 16S rDNA gene sequence played a vital role which is evident by many workers (Al-Ansari et al., 2020, Daigham and Mahfouz, 2020). In current study the molecular identification of the actinomycetes isolate (BHPL**-**KSKU5) was studied, and it was found that Streptomyces sp, which is found 98% similarity with the existing species of *Streptomyces felleus*.

Antimicrobial compounds normally produced from plant ingrediants, but nowadays which also produced from soil microbes such as Actinomycetes; it was great intrest due to which acting as a potent alternative antibacterial agents (Putri et al., 2020). The present study revealed that *S. felleus* (BHPL**-**KSKU5) could produce antimicrobial compounds in starch casein broth. Vijayakumar et al., (2012), reported a similar type of antimicrobial activity against different bacteria using marine actinomycetes isolated from the east coast region of India. Budhathoki and Anima (2020) reported the soli sample isolated actionomyceties from Nepal showed good antibacterial activity against gram positive and negative bacteria, compare to this study present investigation showed good antibacterial activity. The other study Kumar et al. (2021) reported the actinomycetes isolated from arid zone of Thar desert India, purified bioactive compounds showed good antibacterial activity against human pathogenic bacteria.

Further, studies need to be done to purify, charecterize the antimicrobial compound and its optimization for large-scale production. Therefore, the present study suggests that the diversity of antimicrobial producing actinomycetes in coalmine soils of Telangana were predominantly more. The phenotypic, genotypic characterization of the antimicrobial compound from *S. felleus* was done. Hence, coalmine soil is the best suitable source for the isolation of diverse actinomycetes.

## 5 Conclusions

Actinomycetes are the highest microorganisms with the potential to produce novel antibiotics as industrially important secondary metabolites. Actinomycetes could be found in different environments such as soil, husk, and other than the source and our preferred site of coalmine soils are more tolerant of their growing and novel antibiotics and significant secondary metabolites. In present study totally 28 coalmine soil samples were collected from different districts in Telangana. The novel strain BHPL-KSKU5 was identified and characterized as *S. felleus,* which contain more antibacterial and antifungal activity. Further, this study may be helpful to develop potential antibacterial and antifungal agents against different pathogens.

## Declaration of Competing Interest

The authors declare that they have no known competing financial interests or personal relationships that could have appeared to influence the work reported in this paper.
